# Tissue engineering of corneal stroma with rabbit fibroblast precursors and gelatin hydrogels

**Published:** 2008-10-03

**Authors:** Tatsuya Mimura, Shiro Amano, Seiichi Yokoo, Saiko Uchida, Satoru Yamagami, Tomohiko Usui, Yu Kimura, Yasuhiko Tabata

**Affiliations:** 1 Department of Ophthalmology, University of Tokyo Graduate School of Medicine, Tokyo, Japan; 2 Department of Corneal Tissue Regeneration, University of Tokyo Graduate School of Medicine, Tokyo, Japan; 3 Department of Biomaterials, Field of Tissue Engineering, Institute for Frontier Medical Sciences, Kyoto University, Kyoto, Japan

## Abstract

**Purpose:**

To isolate fibroblast precursors from rabbit corneal stroma using a sphere-forming assay, to engineer corneal stroma with the precursors and gelatin, and to establish the therapeutic application of precursors in a rabbit corneal stroma.

**Methods:**

In the in vitro study, a sphere-forming assay was performed to produce precursors from rabbit corneal stroma. Corneal stroma was engineered by cultivating precursors in porous gelatin for one week. In the in vivo study, the engineered corneal stromal sheet with precursors (precursor/gelatin group) or with fibroblasts (fibroblast /gelatin group) or without cells (gelatin group) was transplanted to a pocket of rabbit corneal stroma. Gene expression and extracellular matrix production were examined immunohistochemically in each group one week and four weeks after surgery.

**Results:**

In the in vitro study, cells in the spheres were BrdU-positive, and their progeny were keratocan-positive. The study also showed that the corneas transplanted with a porous gelatin sheet did not show any opacity four weeks after transplantation in any group. In the gelatin sheet of the precursor/gelatin group, a more intense expression of type I collagen was observed relative to the other two groups four weeks after the surgery.

**Conclusions:**

Our findings demonstrate that the transplantation of fibroblast precursors combined with gelatin hydrogel into the corneal stroma is a possible treatment strategy for corneal stromal regeneration.

## Introduction

Although corneal transplantation has achieved clinical success, there is a shortage of corneal donors worldwide. To solve this problem, researchers have attempted to tissue-engineer the cornea. Among the three components of the cornea (epithelium, stroma, and endothelium), tissue-engineered corneal epithelial cell sheets have been used clinically to create the corneal epithelium in patients with total limbal stem cell deficiencies [1-4]. Tissue-engineered corneal endothelial cell sheets have successfully restored corneal transparency in animal models [5-10] and are ready to be used in clinical cases. In contrast, none of tissue-engineered corneal stroma have been deemed clinically feasible, although several kinds of tissue-engineered corneal stroma have been reported [11].

For successful tissue engineering, stem cells and progenitor or precursor cells, which possess proliferative capacity and multilineage developmental potential, are proposed to be an effective cell source. Regenerative stem cells or precursors can be detected in various adult tissues with a sphere-forming assay, including the central nervous system [12], bone marrow [13], skin [14,15], retina [16], and corneal endothelium [8-10,17-20]. Stem and progenitor cells retain their regenerative potential and may promote rapid wound healing compared with differentiated fibroblasts after they are transplanted to recipients. The use of adult stem cells or precursors presents an ideal strategy for tissue regeneration and engineering [21]. Despite these many successes in the isolation and characterization of stem cells from various tissues, relatively few studies have investigated the efficacy of stem cell transplantation therapy. Using a sphere assay, we succeeded in isolating precursor cells from human and rabbit corneal stroma [20,22,23]. In this study, we have constructed a substitute for corneal stroma using fibroblast precursors and porous gelatin hydrogels in vitro by tissue engineering and have investigated the feasibility of transplantation of the engineered corneal stroma in a rabbit model.

## Methods

### Sphere-forming assay

Rabbits were treated in accordance with the ARVO Statement on the Use of Animals in Ophthalmic and Vision Research. Sixteen corneas were excised from eyes of eight New Zealand White rabbits weighing 2.0–2.4 kg (Saitama Experimental Animals Inc., Saitama, Japan) under deep anesthesia. The entire corneal epithelium and endothelium with Descemet’s membrane were mechanically removed. The stroma was cut into small pieces approximately 1.0 mm in diameter and the pieces were incubated overnight at 37 °C in basal medium containing 0.02% collagenase (Sigma-Aldrich, St. Louis, MO). Subsequently, the tissue pieces were dissociated into single cells with 0.2% EDTA. Dissociated single cells were used for sphere-forming assay or culture of fibroblast. The basal medium was composed of Dulbecco’s modified Eagle’s medium (DMEM)/F12 supplemented with B27 (Invitrogen, Carlsbad, CA), 20 ng/ml epidermal growth factor (EGF; Sigma-Aldrich), and 40 ng/ml basic fibroblast growth factor (bFGF; Sigma-Aldrich). The sphere-forming assay was used for primary cell culture [24,25]. Cells were cultured in the basal medium containing a methylcellulose gel matrix (0.8%; Wako, Osaka, Japan) at a density of 10 viable cells/μl in the uncoated wells of 60 mm culture dishes. For passaging, primary spheres (collected on day 7) were dissociated into single cells with 0.5% EDTA, and cells were cultured at a density of 10 cells/μl in basal medium containing 0.8% methylcellulose.

### Differentiation of sphere colonies

Individual primary spheres (collected on day 7) were transferred to 13 mm glass coverslips coated with 50 μg/ml poly-L-lysine (PLL; Sigma-Aldrich, Tokyo, Japan) and 10 μg/ml fibronectin (BD Biosciences, Billerica, MA) after each glass coverslip was put in 24 wells. To promote differentiation, 1% fetal bovine serum (FBS) was added to the basal medium, and the culture was continued for another seven days.

### Culture of corneal fibroblast

Isolated fibroblasts were counted with a hemocytometer. Dissociated single primary fibroblasts were plated at a density of 5.0×10^4^ cells/ml in a 60 mm tissue culture dish and cultured in DMEM (Sigma-Aldrich) supplemented with 10% FBS (Sigma-Aldrich) for seven days.

### Immunocytochemistry of keratocan in spheres and progenies

Immunocytochemical examination of the seven-day spheres and their progeny was performed after seven days of adherent culture on the glass coverslips. Cells were fixed with 4% paraformaldehyde (Wako Pure Chemical Industries, Osaka, Japan) in phosphate-buffered saline (PBS) for 10 min. After washing in PBS, the cells were incubated for 30 min with 3% bovine serum albumin (BSA; Sigma-Aldrich) in PBS, which contained 0.3% Triton X-100 (BSA/TBST; Rohm & Haas, Philadelphia, PA) to block nonspecific binding. Next, the cells were incubated for 2 h at room temperature with the following primary antibodies diluted in BSA/PBST: goat anti-keratocan polyclonal antibody (1:400; Santa Cruz Biotechnology, Santa Cruz, CA) and FITC-conjugated mouse anti-5-bromo2’-deoxyuridine (BrdU)/fluorescence mAb (1:100; Roche Diagnostics, Basel, Switzerland). After being washed in PBS, the cells were reacted for 1 h at room temperature with fluorescence-labeled goat anti-rabbit IgG (Alexa Fluor 594, 1:400; Molecular Probes, Eugene, OR) as the secondary antibodies for the anti-keratocan antibody. Finally, fluorescence was detected by observation under a fluorescence microscope (model BH2-RFL-T3 and BX50; Olympus, Tokyo, Japan).

### Preparation of gelatin hydrogels and electron microscopic observation

Porous gelatin hydrogels were prepared through the chemical cross-linking of aqueous gelatin solution with glutaraldehyde according to the method described elsewhere [26,27]. Briefly, an aqueous gelatin solution mixed with glutaraldehyde was cast into a polypropylene dish followed by the cross-linking reaction at 4 °C for 12 h. The hydrogel samples were stirred in 100 mM aqueous glycine solution at 37 °C for 1 h and washed with double-distilled water. Following the washing with sterilized saline, the hydrogel samples were trephined with a 5.0 mm diameter trephine (Kai Medical, Gifu, Japan). The structure of the gelatin hydrogel was observed using scanning electron microscopy.

### Sphere or fibroblast seeding onto gelatin hydrogels and cell culture

Ten primary fibroblast spheres (50 μm or more in diameter) cultured for seven days or 100,000 primary cultured fibroblasts were applied to the gelatin hydrogels after which the gelatin hydrogels were placed in 5.0 mm diameter wells and centrifuged at 1,000 rpm (176x g) for 10 min to promote the attachment of cells to gelatin hydrogels. The tissue-engineered samples were then maintained in the basal medium containing 10% FBS in a 24 well culture dish for seven days after which the medium containing debris was removed. The primary spheres (50 μm of the diameter) contained approximately 300 cells. In some experiments, primary spheres were labeled with a fluorescent cell tracker (CM-DiI; C-7000; Molecular Probes) to trace their localization as described elsewhere [7].

### Transplantation of gelatin hydrogels with corneal fibroblast precursors

**Figure 1:**
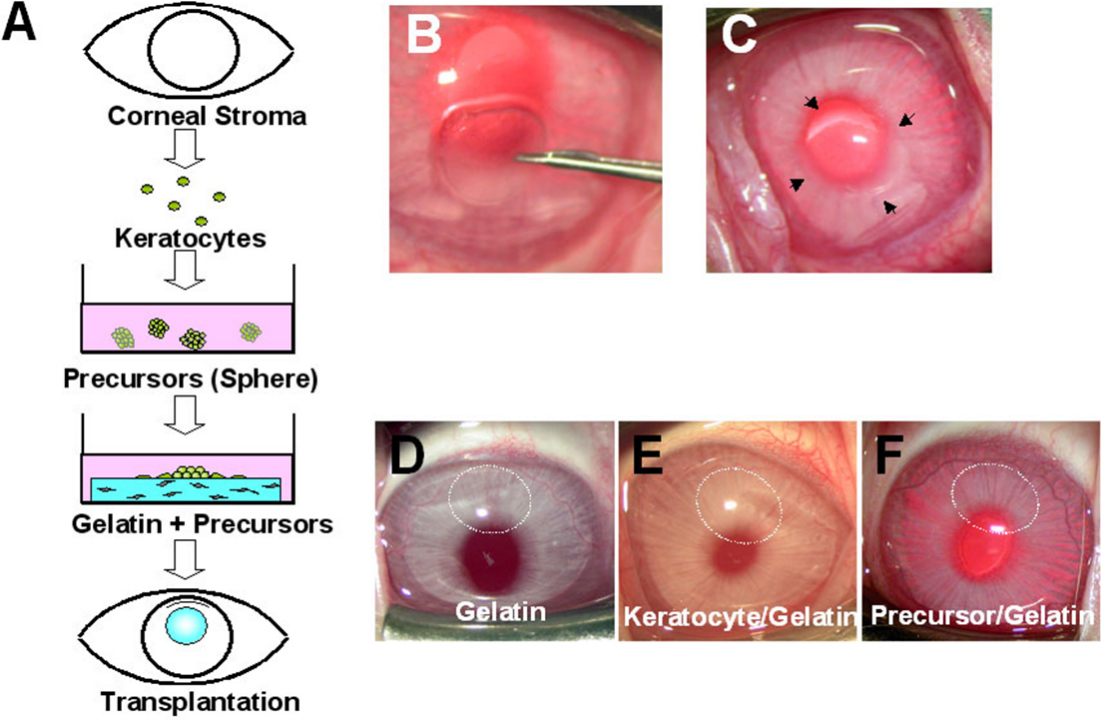
Schematic illustration and clinical findings. **A**: Fibroblast precursors were isolated from the rabbit corneal stroma using a sphere-forming assay. Corneal stroma was engineered by cultivating precursors in porous gelatin for one week. The engineered corneal stromal sheet with precursors was transplanted in a pocket of rabbit corneal stroma. **B**,**C**: Gelatin hydrogels (gelatin group), gelatin hydrogels with corneal fibroblasts (fibroblast/gelatin group), or gelatin hydrogels with corneal fibroblast precursors (precursor/gelatin group) were implanted into the corneal stroma (indicated by arrows in **C**). **D**-**F**: Representative photographs of corneas four weeks after transplantation in each group are shown. No corneal opacity and no rejection were observed in any group four weeks after transplantation (indicated by dotted white circles in **D**-**F**).

A lamellar dissection was made in 24 eyes of 24 rabbits (Figure 1A,B). The engineered corneal stroma was implanted into a mid-stroma corneal pocket without suture fixation. Twenty-four rabbits were divided into three groups, the gelatin group (n=8) transplanted with gelatin hydrogels alone, the fibroblast/gelatin group (n=8) transplanted with fibroblasts cultured on gelatin hydrogels, and the precursor/gelatin group (n=8) transplanted with fibroblast precursors cultured on gelatin hydrogels.

### Histological examination and immunohistochemistry of extracellular matrix

One week (n=2 in each group) or four weeks (n=6 in each group) after transplantation, the rabbits were sacrificed with an overdose of intravenous pentobarbital sodium (Dainippon Pharmaceutical, Osaka, Japan). Each cornea was excised and bisected. The divided corneas or the seven-day cultured gelatin hydrogel sheets for transplantation were fixed in 4% paraformaldehyde, embedded in OCT compound (Tissue-Tek^®^; Miles Laboratories, Naperville, IL), and then frozen at −20 °C. Cryostat sections were cut into pieces 6 µm thick and stained with hematoxylin and eosin. For immunohistochemical staining, the sections were treated with 3% hydrogen peroxide in PBS (Sigma-Aldrich) for 15 min and then rinsed in PBS. After incubation with normal goat serum for 10 min at room temperature, tissue sections were incubated with primary antibodies (goat anti-rabbit type I collagen, type IV collagen, laminin, and vimentin polyclonal antibodies; all from Santa Cruz Biotech) at a concentration of 3 μg/ml overnight at 4 °C. Immunoreactivity was detected by the streptavidin-biotin-peroxidase method using a Histofine SAB-PO kit (Nichirei Corporation, Tokyo, Japan). The final reaction product was visualized using 3,3′-diaminobenzidine tetrahydrochloride.

### Immunohistochemistry of CD34 and nestin

In the precursor/gelatin group, frozen sections (8 μm) were stained with mouse anti-CD34 mAb (1:100; Novocastra Laboratories Ltd., Newcastle upon Tyne, UK) or mouse anti-nestin mAb (1:400; BD Biosciences, San Jose, CA) for 2 h and incubated with fluorescence-labeled goat anti-mouse IgG (Alexa Fluor 488, 1:200; Molecular Probes) for 30 min at room temperature. After several washings with PBS, the sections were coverslipped using anti-fading mounting medium (Vectashield; Vector Laboratories, Burlingame, CA) and were observed under the fluorescein microscope.

## Results

### Isolation of sphere colonies and secondary sphere formation

**Figure 2:**
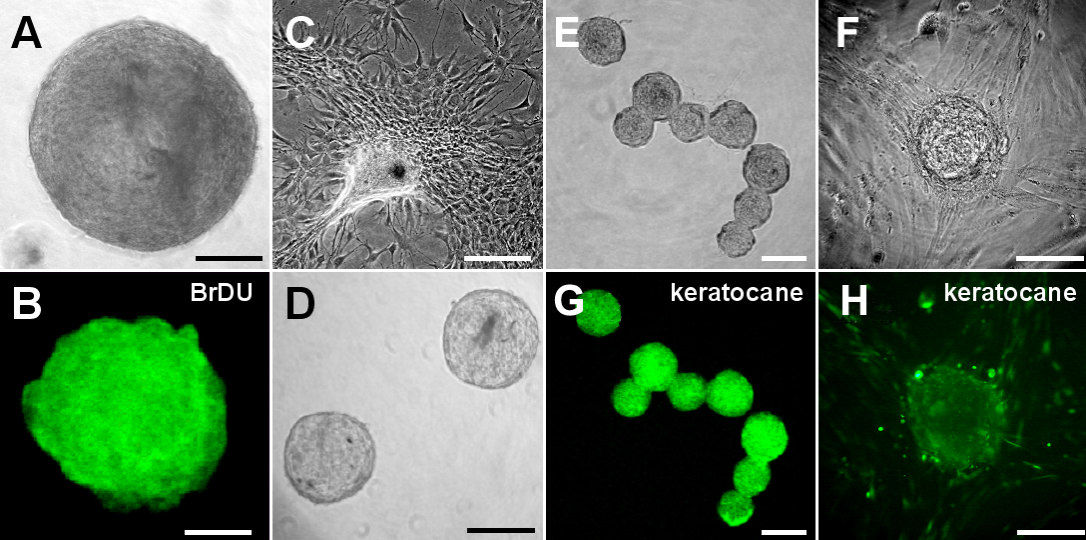
Sphere formation from rabbit corneal fibroblasts. **A**: A representative day 7 sphere from rabbit corneal fibroblasts had a diameter of approximately 300 μm. **B**: Each primary sphere was positive for BrdU on day 7. **C**: The differentiated progeny from the primary sphere showed a typical fibroblast-like morphology. **D**: Secondary spheres were generated from dissociated primary spheres. **E** and **F**: Day 7 spheres were positive for keratocan. **G** and **H**: Progenies derived from the sphere are positive for keratocan. Scale bar=100 μm.

Corneal stroma was disaggregated into single cells, which were cultured for seven days. A photograph of a representative sphere is shown in Figure 2A. Many cells within the sphere colonies were positive for BrdU, indicating active DNA synthesis (Figure 2B). The cells from primary spheres primarily differentiated into fibroblast-like cells (Figure 2C). Secondary spheres were generated from dissociated primary spheres (Figure 2D). The primary spheres’ diameters are larger than those of the secondary spheres as shown in Figure 2A,D. Additionally, replating to generate secondary sphere colonies was less efficient than the generation of primary spheres [23]. These results indicate that the precursor cells had a limited proliferative capacity.

### Immunocytochemical analysis of extracellular matrix in gelatin hydrogels

**Figure 3:**
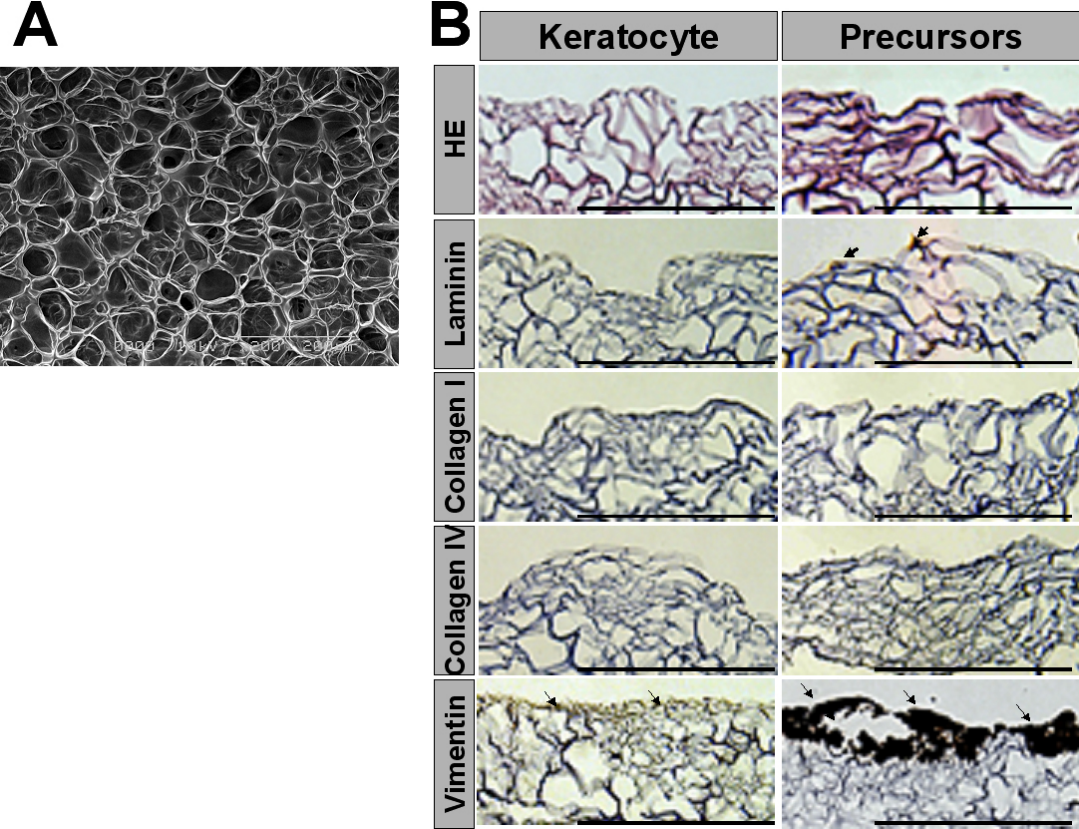
Immunohistochemical analysis of extracellular matrix in porous gelatin hydrogels with corneal fibroblasts or fibroblast precursors. Corneal fibroblasts or fibroblast precursors were seeded onto porous gelatin hydrogels and cultured for one week. **A**: Scanning electron microscopy revealed a porous structure for the gelatin hydrogels. **B**: Hematoxylin and eosin staining and immunohistochemical analysis of vimentin and ECM in porous gelatin hydrogels seven days after seeding of corneal fibroblasts or fibroblast precursors are shown. Vimentin staining was more intense in the gelatin hydrogels with corneal fibroblast precursors than in those with corneal fibroblasts (arrows). Other ECM components such as laminin, type I collagen, and type IV collagen are not expressed in the gelatin hydrogels with corneal fibroblasts or fibroblast precursors before transplantation except for a weak expression of laminin in the gelatin hydrogels with corneal fibroblast precursors (arrow). Scale bar=200 μm in **B**.

Scanning electron microscopy demonstrated porous structure of the gelatin hydrogels (Figure 3A). Corneal fibroblasts or fibroblast precursors were cultured on porous gelatin hydrogels for seven days, and the expression of vimentin, a marker of mesenchymal cells, as well as extracellular matrix (ECM) production were evaluated. The positive staining with vimentin suggests that the progenitor cells were not converted into inflammatory cells. Vimentin expression was more intense in porous gelatin hydrogels with fibroblast precursors than it was in those with corneal fibroblasts (Figure 3B). Weak expression of laminin was also seen in the gelatin hydrogels with fibroblast precursors while no expression was observed in those with corneal fibroblasts (Figure 3B). Little expression of types I and IV collagen was detected in the porous gelatin hydrogels with corneal fibroblasts or fibroblast precursors (Figure 3B).

### Clinical observation after surgery

The therapeutic use of precursors derived from corneal fibroblasts was investigated in a rabbit model (Figure 1A-C). As shown in the representative anterior segment photographs from the gelatin group (transplanted with gelatin hydrogels alone, Figure 1D), the fibroblast/gelatin group (transplanted with gelatin hydrogels with corneal fibroblasts, Figure 1E), and the precursor/gelatin group (transplanted with gelatin hydrogels with corneal fibroblast precursors, Figure 1F), the corneas were clear and showed no edema and no cell infiltration of the stroma in all of the groups. No apparent immunological reactions were observed during the follow-up period. No side effects including an increase in intraocular pressure, corneal neovascularization, corneal ulcer, or corneal infection were detected during the observation period.

### Histological examination and analysis of extracellular matrix production with immunohistochemistry

**Figure 4:**
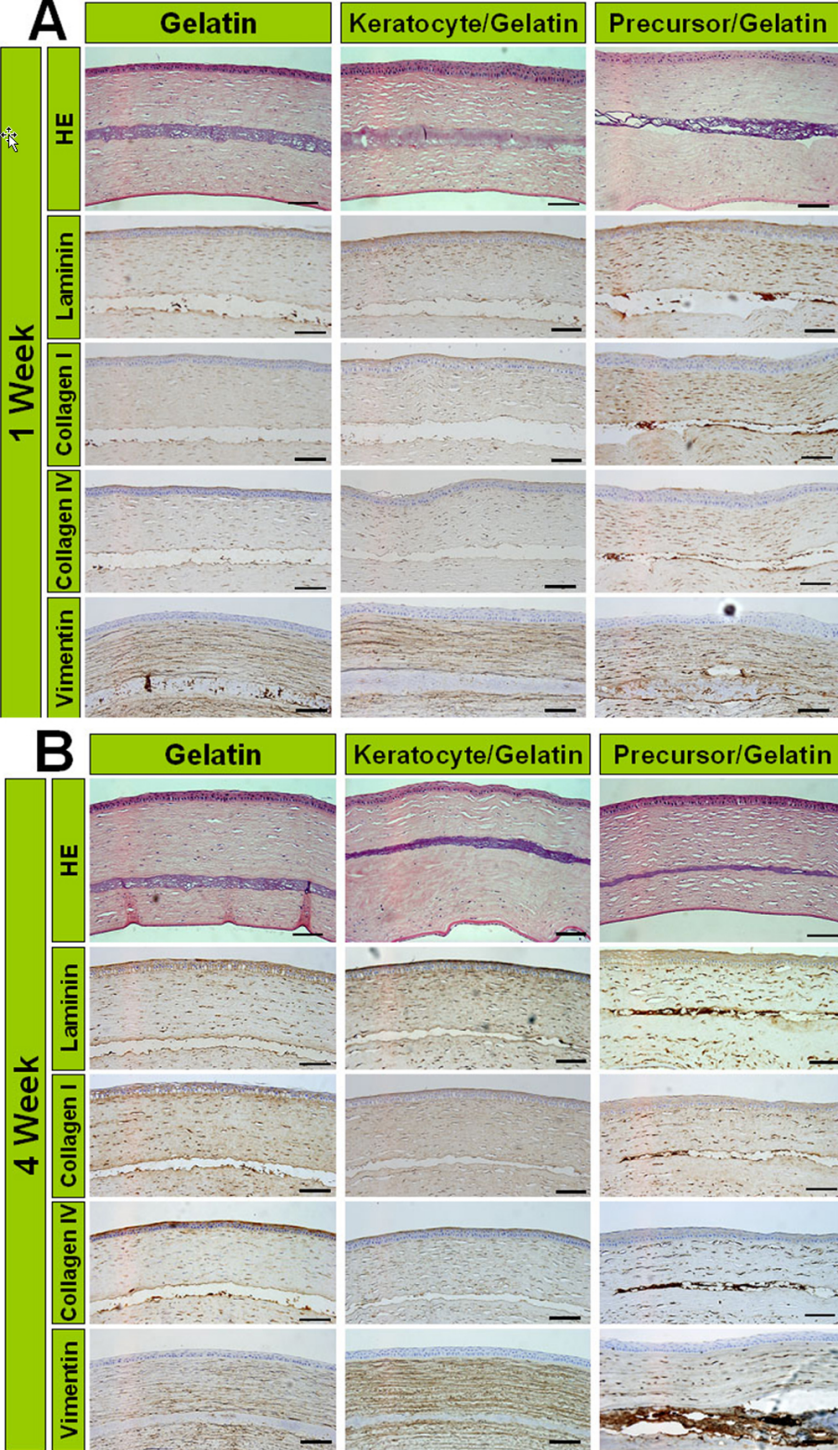
Histological findings and immunocytochemical analysis of extracellular matrix at one week and four weeks after transplantation. The transplanted gelatin hydrogels are found in the corneal stroma in all groups. H&E staining reveals no mononuclear cell infiltration around gelatin hydrogels in all groups. The precursor/gelatin group shows more intense staining of laminin, type I collagen, type IV collagen, and vimentin in the transplanted gelatin than in the gelatin and fibroblast/gelatin groups one week after transplantation (**A**). These expressions in the precursor/gelatin group increase four weeks after transplantation (**B**). Scale bar=100 μm.

The corneas were excised from the eyes either one week or four weeks after transplantation. The expressions of vimentin and ECM molecules such as laminin, type I collagen, and type IV collagen were more intense in the precursor/gelatin group than the other groups after one week (Figure 4A). Their expression increased after four weeks in the precursor/gelatin group (Figure 4B).

### Expression of progenitor cell markers in the transplanted gelatin hydrogels

**Figure 5:**
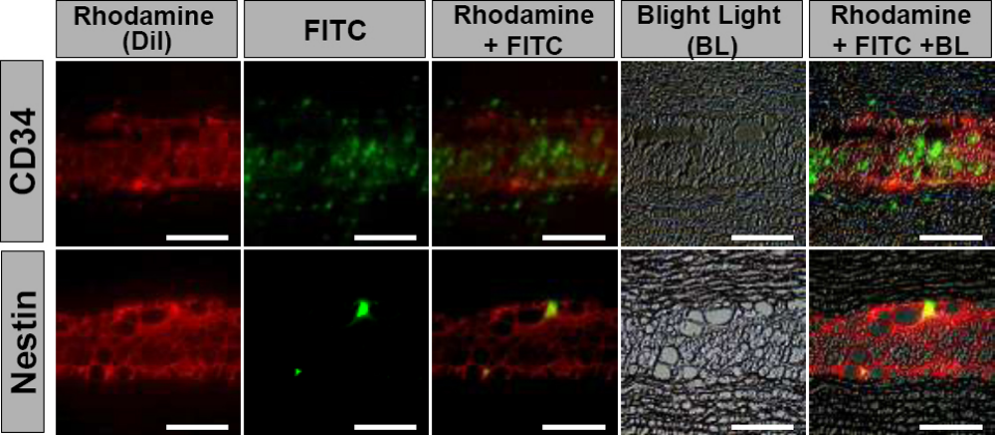
Immunolocalization of CD34-positive or nestin-positive cells within the transplanted DiI-positive precursors in the precursor/gelatin group four weeks after transplantation of gelatin hydrogels with corneal fibroblast precursors. Bright light (black and white, background), rhodamine (red, the transplanted DiI-labeled corneal fibroblast precursors in the gelatin hydrogels), and FITC (green, CD34- or nestin-positive cells) are superimposed with Adobe Photoshop software. Whole transplanted gelatin hydrogels are shown in light red by many DiI-positive corneal fibroblast precursors. A few CD34-positive cells or nestin-positive spindle cells are scattered within the gelatin hydrogels. Scale bar=100 μm.

We examined the expression of CD34, which is a well known hematopoietic stem/progenitor cell marker and is also expressed by all differentiated keratocytes [28], and nestin, a neural progenitor cell marker in the precursor/gelatin group, four weeks after transplantation. Many CD34-positive cells were detected in and around the transplanted gelatin hydrogels (Figure 5A). Nestin-positive cells were also observed in the transplanted gelatin hydrogels (Figure 5B). No CD34-positive or nestin-positive cells were seen in the other groups in the transplanted gelatin hydrogels (data not shown).

## Discussion

We established the therapeutic application of precursors in a rabbit corneal stroma using fibroblast precursors and gelatin hydrogels as the substitute carrier of corneal stroma. Cultured precursors derived from adult rabbit stroma retain the essential fibroblast morphology. The transplanted gelatin hydrogels with fibroblast precursors induced the production of ECM by the host stroma.

Adult vertebrate corneal stroma is composed primarily of collagen type 1 fibrils, smaller amounts of other ECM proteins, and keratocytes. Therefore, both cells and a stratified complex of the ECM, which form a scaffold for precursors, are necessary to engineer a three-dimensional corneal stroma. Porous scaffolds used in tissue engineering contribute to cell proliferation and differentiation in a suitable environment as well as the maintenance of structure and composition in the injured tissues. Furthermore, three-dimensional porous scaffolds provide a larger surface for cell attachment, migration, and proliferation compared with two-dimensional scaffolds, which facilitate contact inhibition in confluent cells. Because corneal fibroblast proliferation is substrate-dependent, it is preferable to increase the surface area of the culture substrate. Several three-dimensional substrates such as collagen have been designed to demonstrate their capacity for proliferative enhancement [29-31]. Their long-term safety, stability, and efficacy in vivo have been adequately established in humans. However, the significant drawback of collagen is its poor biodegradation and bioabsorption. The biodegradation of biomaterials and their biocompatibility are probably their most desired properties because the insoluble biomaterials develop biological reactions in vivo resulting in tissue opacity. Gelatin, a denatured type of collagen, possesses most of the properties of an ideal scaffold and has been applied clinically as an implant material [32]. We used a biodegradable porous gelatin hydrogel, which is effective in facilitating cell migration and the delivery of oxygen and nutrients to the migrated cells, as a carrier of corneal fibroblast precursors. In the previous in vivo degradation tests in the conjunctival sac of mice, the residual radioactivity in carrier gels ranged from 50.7% to 54.7% on day 1 and from 11.2% to 18.4% on day 7. [33]. In monkey skull defect models, the gelatin hydrogel with a water content of 93.8 wt% did not degrade and remained at the skull defect site 12 weeks after application [34]. In the current study, the hydrogel did not degrade in the corneal stroma four weeks after transplantation. The rate of hydrogel degradation in the corneal stroma may be slow compared with those of the conjunctival sac of mice and skull defect models of the monkey.

Each primary sphere (50 μm in diameter) contained approximately 300 cells. Therefore 10 spheres contain approximately 3,000 cells. Despite the total cell number within 10 corneal fibroblast spheres being approximately 30 fold less than 100,000 corneal fibroblasts, the porous gelatin hydrogels that incorporated 10 corneal fibroblast spheres cultured ex vivo for seven days showed more intense immunostaining for vimentin than those incorporating 100,000 corneal fibroblasts cultured for seven days, which indicates that the corneal fibroblast precursors had superior proliferative potential on the gelatin hydrogels compared with corneal fibroblasts. Where weak expression of laminin and collagens were detected in gelatin hydrogels that incorporated corneal fibroblasts precursors, these ECM components were barely detected in the gelatin incorporating corneal fibroblasts ex vivo before transplantation. These results indicate that the gelatin hydrogel itself had no ability to induce tissue regeneration in vitro or ex vivo.

We have previously produced spheres from human [22] and rabbit [23] corneal stroma. The individual spheres and their progeny expressed mesenchymal and neuronal lineage marker proteins. Moreover, they expressed keratocan that were used as keratocyte-specific markers [35-37], suggesting that fibroblast progenitors and progenies have an essential lineage of keratocytes. Immunohistochemical findings on day 7 and 28 showed that the expression of type I collagen, type IV collagen, laminin, and vimentin were strongly positive in the transplanted gelatin hydrogels of the precursor/gelatin group while they were faint in the gelatin and fibroblast/gelatin groups. Gelatin hydrogels alone function to induce ECM production to some extent in vivo, but the efficacy is not as high as that of the gelatin hydrogels with precursors.

In the ex vivo experiments, the expression of ECMs were low in all groups while the in vivo experiments showed that vimentin expression in transplanted gelatin hydrogels was higher in precursor/gelatin groups compared with the other two groups. In Figure 4A, the corneal stroma as a whole (even tissue far away from the transplanted tissue) had stronger expression of vimentin and ECM in the precursor/gelatin group than the other groups. Also in Figure 4A, vimentin expression in the gelatin group was stronger than that in the fibroblast/gelatin group. These suggest that ECM and vimentin arise from the host and not the transplanted cells.

Furthermore, expression of ECM and vimentin increased four weeks after transplantation in all groups compared with just one week. The gelatin only also promotes ECM secretion. Both precursors and gelatin may promote the production of ECM derived from the host stroma and not the transplanted cells.

Immunofluorescence microscopy of the precursor/gelatin group on day 28 revealed that the CD34-positive cells and nestin-positive cells were localized to the transplanted gelatin hydrogels. This indicates that fibroblast precursors with a greater self-renewal potential continue to proliferate even after transplantation and can supply fibroblasts necessary for the regeneration of the host stroma. The nestin-positive cells may also contribute to induction of nerve regeneration.

The combined transplantation of corneal fibroblast precursors with gelatin hydrogels into a corneal stromal pocket has several advantages over penetrating keratoplasty of full-thickness donor cornea. Most complications associated with open-sky surgery such as expulsive hemorrhage and the risks of wound dehiscence would be eliminated. Several postoperative complications such as a postoperative corneal irregular astigmatism, wound leakage, corneal infection, vascularization, and persistent epithelial defect can be avoided. Allograft rejection is a leading cause of the failure of conventional full thickness corneal allografting with local and/or systemic immunosuppressants. Histologically, no apparent inflammatory reaction including immunological rejection was detected in the current corneal fibroblast precursor allotransplantation. This finding suggests that the transplanted precursors can survive in the corneal stroma without rejection.

One of the major limitations for clinical application of corneal fibroblast precursor transplantation is the availability of donor fibroblast precursors in sufficient quantities. This study demonstrated that the required number of spheres per cornea was 10 spheres to promote the expression of ECM. In the human cornea, the number of digested stromal cells from individual corneas was 1.1×10^5^, and the number of spheres grown from one cornea was 1,566±211 [22], which are adequate quantities for transplantation. These results suggest the feasibility of transplantation of autologous fibroblast precursor derived from a small piece of stroma. The main weakness of this study may be the relatively short observation period. Further long-term investigation including side effects, rejection, corneal opacity, infection incidence, and histological observation is necessary. Additionally, our tissue engineering based on the ECMs, which were not abundant in normal cornea, does not represent restoration of a true stromal ECM. Further investigation is necessary to show stromal-specific matrix molecules such as keratan sulfate or keratocan in the transplanted gelatin hydrogels.

As for the in vivo physiologic function of the transplanted cornea, we could not perform the corneal sensitivity test in the animal model because we cannot evaluate the subjective corneal sensitivity of an animal using a Cochet-Bonnet esthesiometer, which is the only corneal sensitivity test. Although we had no idea of corneal stress test, we found that all groups had no obvious corneal weakness and there was no significant difference in intraocular pressure among the three groups. As for corneal nerves in the host tissues, a microscopic examination of the frozen section and a confocal microscopic examination of whole mount cornea showed no distinguished corneal nerve in the host cornea after transplantation because it is generally difficult to observe the corneal nerve. Further examinations about corneal physiologic function after precursor transplantation may be necessary.

In summary, we established a method for three-dimensional tissue engineering of the substitute for corneal stroma using fibroblast precursors and gelatin hydrogels. Corneal fibroblast precursors-based corneal stromal regeneration combined with gelatin hydrogel is a promising new therapy to promote fibroblast adherence and ECM deposition after corneal fibroblast precursor transplantation. This therapeutic tissue engineering is applicable to any type of cell in regenerative medicine. The transplantation of corneal fibroblast precursors into a corneal stromal pocket proved to be a simple and effective treatment strategy for corneal regeneration, which may replace conventional full-thickness corneal grafting and compensate for the worldwide shortage of donor corneas.
